# Effects of comprehensive sexuality education on the comprehensive knowledge and attitude to condom use among first-year students in Arba Minch University: a quasi-experimental study

**DOI:** 10.1186/s13104-019-4746-6

**Published:** 2019-10-26

**Authors:** Negussie Boti, Sultan Hussen, Mulugeta Shegaze, Simon Shibru, Tamiru Shibiru, Eshetu Zerihun, Wanzahun Godana, Sintayehu Abebe, Woyinshet Gebretsadik, Nathan Desalegn, Zebene Temtime

**Affiliations:** 1 College of Medicine & Health Sciences, Department of Public Health, Arba Minch University, Arba Minch, Ethiopia; 2 College of Medicine & Health Sciences, School of Medicine, Arba Minch University, Arba Minch, Ethiopia; 3 College of Natural Sciences, Department of Biology, Arba Minch University, Arba Minch, Ethiopia; 4 College of Medicine & Health Sciences, Department of Nursing, Arba Minch University, Arba Minch, Ethiopia; 5 Department of Psychology, College of Social Sciences and Humanities, Arba Minch University, Arba Minch, Ethiopia

**Keywords:** Comprehensive sexuality education, Knowledge to condom, Attitude to condom: intention to condom use, University student: Ethiopia

## Abstract

**Objective:**

To assess the effect of comprehensive sexuality education on the comprehensive knowledge and attitude to condom use among first-year students at Arba Minch University.

**Results:**

A total of 832 students participated at a baseline, and 820 students participated at the posttest. This study found that there was a significant effect on changing students’ knowledge and attitude towards a condom. In the education group, the students’ average change of comprehensive condom knowledge score was 0.229 higher than the average score of students’ in the control group (ATE = 0.229, 95% CI 0.132 to 0.328; p < 0.001). The average change of attitude toward condom score of students’ in the education group was 1.834 higher than the average change score of students’ in the control group (ATE = 1.834, 95% CI 1.195 to 2.772; p < 0.001).This study provides further evidence on the effectiveness of comprehensive sexuality education in terms of knowledge and attitude towards a condom. Therefore, the implementation of this education should be strengthened in order the prevent youths from STI/HIV and unintended pregnancies.

## Introduction

According to the world health organization (WHO) Youth is an important population group with great potential for physical, mental, and psychological development [1]. Youths are the largest population there were 1.80 billion people between the ages of 10 and 24 years, of the 70% are concentrated in developing countries [2]. In Ethiopia from the total population, 20.04% were between 15 and 24 years [3, 4]. Most of them are joining a higher institution for an academic program [5, 6]. Higher education institutions in Ethiopia host young people aged between 19 and 24 years [7, 8].

According to reports shows that worldwide youths were at high risk of HIV infection, accounting for 20% of new HIV infections [7, 9]. Seventy-nine percent of these infections occur in Sub-Saharan Africa (SSA) [7]. Also, each year 7.4 million girls experienced unintended pregnancies and 3 million girls experienced unsafe abortions [10]. These problems put young people at risk for morbidity, mortality and limiting their educational and employment opportunities [11, 12].

Evidence shows that higher education institutions are the best place to deal with sexual reproductive health problem including HIV/AIDS and unintended pregnancy [11]. Ethiopia started sexual and reproductive health prevention information and services accessible to higher learning institutions Since 2008 [2, 4].

Even though, comprehensive sexuality education is implemented in Ethiopia as one of the sexual and reproductive health-related problems prevention and control strategies among higher institution youth in Ethiopia including the study area. However, the effect of this education was not assessed previously in Ethiopia. Therefore, this study aims to assess the effect of an education intervention on knowledge and attitude towards a condom among first-year students of Arba Minch University in 2017/18.

## Main text

### Methods

#### Study design and setting

A quasi-experimental study was conducted among first-year students of Arba Minch University.

#### Population

All first-year students of Arba Minch University in regular programs were the source population whereas all first-year students in selected departments who attend regular programs and full fill inclusion criteria in both intervention and control groups were study population.

#### Inclusion and exclusion criterion (eligibility criteria)

The study included all first-year students whose ages between 15 and 24 years, but those students who had previous exposure for comprehensive sexuality education and those who have unable to respond due to severe illness were excluded from the study.

#### Sample size determination

The required sample size for the number of students needed for this study was calculated by Open Statcalc based on the following assumptions: Based on the study done in Lusaka, Zambia, the proportion of students who used condom consistently and correctly with casual partner last time among control group was 59.1% and among intervention group was 71.4% [13]. At a 95% confidence interval for a two-sided test, power of 80%, a minimum detectable alternative of ± 5%. Accordingly, the calculated sample size was 504 participants. Assuming a study refusal rate of 10% and a design effect of 1.5, a total minimum sample size needed for this study was 504* 0.1 + 504 = 554*1.5 = 832 individuals.

#### Sampling technique

**Fig. 1:**
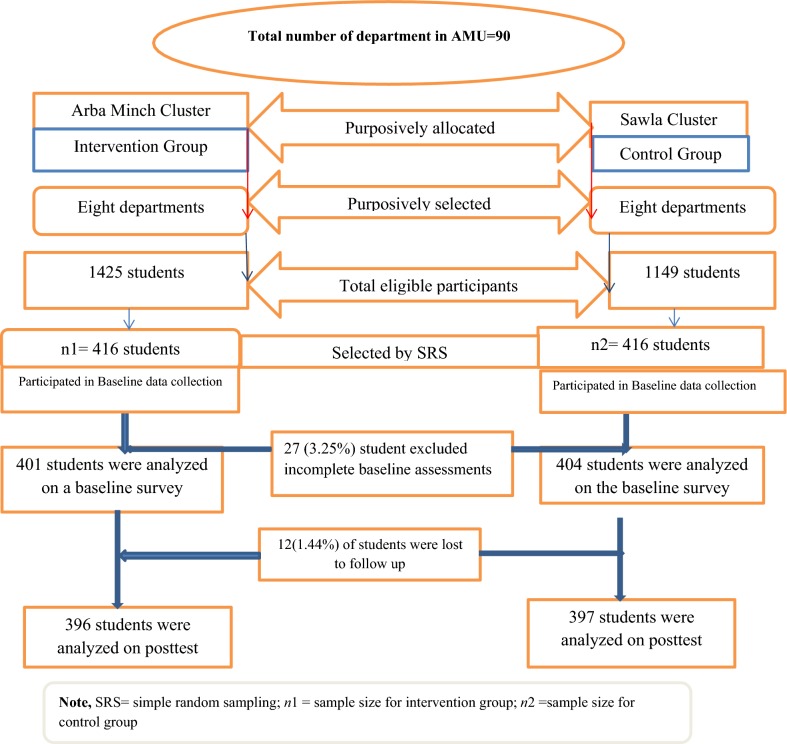
Diagrammatic presentation of sampling procedure for the study on the effects of comprehensive sexuality education on the comprehensive knowledge and attitude to condom use among first-year students in Arba Minch University, 2018

The sampling procedure used in this study was cluster sampling techniques. The sampling frame used for this study was obtained from Arba Minch University registrar office. Arba Minch University geographical located in two towns administrative of zone. The Arba Minch and sawla town. Those towns used as clusters. To select the samples of departments, departments were stratified into two clusters and samples of students were selected independently in each cluster in two stages. In the first stage, a total of 16 departments (8 from each cluster) were selected with purposively and with independent selection in each sampling stratum. In the second stage of selection, a fixed number of 832 (416 per cluster) were selected using simple random sampling techniques using a computer-generated random table (Fig. 1).

#### Measurement

*Knowledge towards condoms* was measured using four items developed after reviewing previously done literature. The scale is comprised of the following question, having heard about the male and female condom, condoms are an effective method to prevent unwanted pregnancy, HIV/AIDS and sexually transmitted infection (STI). The correct answers were coded “1” and wrong answers “0” [14].

*Attitude towards condoms* was measured using thirty Likert scale items developed after reviewing previously done pieces of literature [14–16]. Respondents were presented with the following statements. Response categories included: 1 = agree, 2 = not sure and 3 = disagree.

*Intention to use a condom* was measured based on the study subjects responses to the question asked on regardless of their past sexual experience the students were assessed for their intention to use a condom in their next sexual encounter, using the following item: “I intend to use a condom at the next sexual intercourse.” Responses were arranged from strongly agree to strongly disagree on a 5-point scale. This variable will be treated as continuous and each variable coded as “1” or Have great intention to use condoms If the study subject responded as he/she intend to use a condom at the next sexual intercourse and “0” or student don’t have an intention to use condoms [3, 17].

#### Data collection procedure

Data were collected using a self-administered questionnaire. The questionnaire was initially adopted from the WHO knowledge, attitudes, beliefs, and practices survey instrument and further modified based on available literatures [14, 18–20]. At the pre-intervention stage, the baseline information for respondents was obtained from two groups (intervention and control group) 1 week before starting an educational intervention. The unique confidential identification number was assigned to each student to allow for matching their responses across time points. This was followed by an intervention stage in which a series of 16-week education sessions for students in the intervention group which was designed to equip students with knowledge, skills, and attitudes needed to prevent SRH problems and bring positive behavioral changes on sexual and reproductive issues. The education was delivered using brainstorming, lectures, case study, discussion and demonstrations methods. Immediately, after the last education session before the final exam, the post-intervention stage, the same questionnaire that used in the pre-intervention stage was administered to the same students who were selected at the pre-intervention stage in both the intervention and the control groups.

#### Data quality control

To data quality control training was given for data collectors and data clerk personnel. Intensive supervision was done by investigators during data collection. A pre-test was conducted at Arba Minch Health Science College that was equivalent to 5% of the study participants to check the accuracy and consistency of the data collection tools. All the data were cleaned double entered and cross-checked for their completeness and linkage to the unique identification number before analysis. The database was checked for incorrect or out of range data entry.

#### Data analysis

The completeness and consistency of the data were checked, coded and double entered into Epi-data 3.1 and exported to STATA version 14.0 statistical software for further analysis. Descriptive statistics were performed. Pearson’s Chi squared test was used to compare categorical outcome variables before and after intervention as well as between the intervention and control group. Furthermore, to compare continuous outcome variables before and after the intervention was tested using paired t-tests while differences between the control group and the intervention groups were tested using the independent t-test. To see the effect of intervention we used an inverse probability weighted (IPW) analysis.

### Results

#### Socio-demographic characteristics of the study participants

A total of 416 questionnaires were administered to each study group and control group at the pre-intervention stage of the study. Among those 401 and 404 questionnaires were filled, giving the response rate of 96.75%. At the post-intervention stage, 411 and 409 questionnaires were administered to intervention (education) and control (without education) group, respectively. Among those 396 and 397 questionnaires were filled at this stage giving the response rate of 96.71%. Furthermore, the education and control group were properly matched such that there was no statistically significant difference in their socio-demographic characteristics of respondents (Table 1).

**Table 1:** Socio-demographic characteristics of the study participants in Arba Minch University, Ethiopia, 2018/19

Study group	Variables	Subcategories	Pre-intervention N (%)	Post-intervention N (%)	p-value
Intervention group	Sex	Male	297 (74.1)	294 (74.2)	X^2^ = 0.206 p = 0.694
		Female	104 (25.9)	102 (25.8)	
	Age in year	Mean ± SD	19.55 ± 1.94	19.60 ± 1.14	
	Religion	Orthodox	259 (64.6)	256 (64.6)	X^2^ = 7.39 p = 0.807
		Catholic	2 (0.5)	2 (0.5)	
		Protestant	97 (24.2)	95 (24.0)	
		Muslim	39 (9.7)	38 (9.6)	
		Others	4 (1.0)	5 (1.3)	
	Type of school attended	Governmental	348 (86.8)	347 (87.6)	X^2^ = 42 p = 0.654
		Private	53 (13.2)	49 (12.4)	
	Participating in religion education	Yes	353 (88)	367 (92.3)	X^2=^0.069 p = 1.000
		No	48 (12)	29 (7.3)	
	Ever discussed Sex-related matters	Yes	170 (42.4)	236 (59.6)	X^2^ = 0.796 p = 0.408
		No	231 (57.8)	160 (40.4)	
Control group	Sex	Male	250 (61.9)	245 (61.7)	X^2^ = 0.58 p = 0.595
		Female	154 (38.1)	152 (38.3)	
	Age in year	Mean ± SD	19.67 ± 126	19.69 ± 1.17	
	Religion	Orthodox	278 (68.8)	275 (69.3)	X^2^ = 9.49 p = 0.578
		Catholic	8 (2)	6 (1.5)	
		Protestant	62 (15.3)	61 (15.4)	
		Muslim	54 (13/4)	53 (13.4)	
		Others	2 (0.5)	2 (0.5)	
	Type of school attended	Governmental	351 (86.9)	349 (57.9)	X^2^ = 1.19 p = 0.282
		Private	53 (13.1)	48 (12.1)	
	Participating in religion education	Yes	348 (86.1)	307 (77.3)	X^2^ = 1.44 p = 0.235
		No	56 (13.9)	90 (22.7)	
	Ever discussed Sex-related matters	Yes	168 (41.6)	176 (44.3)	X^2=^0.457 p = 0.539
		No	236 (58.4)	221 (55.7)	

#### Comparison of pre-test and post-test scores in both the education and control groups

From paired t-test analysis, it was found out that there were significant differences in pre-test and post-test mean scores of comprehensive knowledge on condoms of the respondents in the intervention group (p = 0.001). While among the respondents in the control group there are no significant differences in the mean improvement of the scores of Comprehensive knowledge on condoms (p = 0.967). Similarly, finding from paired t-test analysis shows that, there were significant differences in the pre-test and post-test scores of attitude toward condoms (p = 0.006) of the respondents in the intervention group compared to the control group. Also, when comparing the baseline and end-line findings of both the intervention and control groups using Chi square test. The proportions of study participants in the intervention group 148 (36.9%) have the intention to use a condom during pre-intervention and 162 (40.9%) during post-intervention periods (p-value = 0.001).

#### Comparison between education and control groups after intervention

The finding of this study reveals that there is significant difference between education and control group after intervention using independent t-test shows that the mean scores between the two groups related to comprehensive knowledge of condom showed that the intervention group had higher scores than the control group with statistically significant differences (mean diff. = 0.221, 95% CI = 0.12 to 0.32: p = 0.001). Also there is statistically significant differences in students’ attitude to condoms (mean diff. = 2.01, 95% CI = 1.06 to 2.96: p = 0.001).

#### Effect of comprehensive sexuality education on knowledge and attitude to condom

Inverse probability weighting analysis was conducted to see the effect of the comprehensive sexuality education on students’ knowledge and attitude towards condom. All outcome variables were weighted by the baseline characteristics of study participants (sex, age, residence, religion, attendance of religious education and type of school they attended) to reduce the effect of selection bias.

In the education group, the student’s average change of comprehensive condom knowledge score was 0.229 higher than the average score of the student’s in the control group (ATE = 0.229, 95% CI 0.132 to 0.328; p < 0.001). The average change of attitude toward condom score of the student’s in the education group was 1.834 higher than the average change score of students in the control group (ATE = 1.834, 95% CI 1.195 to 2.772; p < 0.001) (Table 2).

**Table 2:** The effect of comprehensive sexuality education on Sexual behavior among first-year Arba Minch University students, Arba Minch, Ethiopia, 2018/19

Variables	Regression coefficient	95% CI	p-value
Comprehensive knowledge about condoms	0.229	(0.132, 0.328)	<0.001*
Attitude toward condoms	1.834	(1.195, 2.772)	<0.001*
Intention to use condoms	0.108	(0.042, 0.175)	0.001*

* p < 0.05 sig. (2-sided)

### Discussion

Results from the current study found that there is a significant difference between education and control group after intervention on student’s comprehensive knowledge and attitude towards condoms. This finding was in line with a study conducted in Los Angeles and Northern Ghana [19, 20]. This study also supported the 2030 Agenda specific all learners acquire knowledge and skills needed to promote sustainable development [21].

Concerning intention to use condoms, the finding of this study that there were significant differences in the changing students’ intention to condom use between the education and control groups after intervention. This finding was supported study conducted in Tanzania, Los Angeles, Zambian and USA [13, 19, 22, 23]. This might be inducted that availing condom around school compound may reduce students from practicing risky sexual behavior that may help the fight against HIV and to Ending the AIDS Epidemic by 2030 [21].

Furthermore, the finding of this study shows that there was no significant difference between the education and control group in the consistent and correct use of condoms after the intervention. This finding was supported by study conduct in Zambian secondary schools which reveal that there was no change in condom use practice after the intervention [13]. But, inconsistent with other studies [14, 24]. Moreover, the previous study suggested that adequate condom related knowledge was not a sufficient determinant to condom use [23]. Furthermore, having information did not have a direct influence on condom use, information indirectly contributed to condom use mediated by behavioral skills [24, 25].

### Conclusion

In conclusion, this study found that comprehensive sexuality education improves the students’ knowledge and had an impact on their attitude and intentions towards condom use. However, the findings of this study did not show a significant effect on students’ consistent and correct use of a condom because this may require time to practice. Therefore, strengthen the implementation of this education should be are necessary for the control and prevention of STI including HIV/AIDS and unintended pregnancy.

## Limitation of the study

This study may have some limitations. First, the campuses were not randomly assigned to intervention and control groups. Although we tried to match some confounding factors such as social–demographic characters, there may be some unknown factors influencing the effect of intervention, which might increase or decrease the real effect of intervention. Second, the intervention period 6 months may not show the long term impact of this program. Thirdly, we use results from a Zambian study as the basis of your sample size calculation as a limitation in the study that may have interfered with the representativeness of our sample.

## Data Availability

The data used to support the findings of this study are available from the corresponding author upon request.

## Abbreviations

AIDS: acquired, immune deficiency syndrome
ATE: average treatment effect
CMHS: College of Medicine and Health Science
HEIs: higher education institutions
HIV: human immuno deficiency virus
IPW: inverse probability weighted
SD: standard deviation
SOP: standard operating procedure
SRH: sexual reproductive health
SRS: simple random sampling
SSA: Sub-Saharan Africa
STI: sexually transmitted infections
WHO: World Health Organization
